# Co-Occurrence of Toxic Bloom-Forming Cyanobacteria *Planktothrix*, Cyanophage, and Symbiotic Bacteria in Ohio Water Treatment Waste: Implications for Harmful Algal Bloom Management

**DOI:** 10.3390/toxins17090450

**Published:** 2025-09-05

**Authors:** Angela Brooke Davis, Morgan Evans, Katelyn McKindles, Jiyoung Lee

**Affiliations:** 1 Division of Environmental Health Sciences, College of Public Health, The Ohio State University, Columbus, OH 43210, USA; angelabdavis@hotmail.com (A.B.D.);; 2 Department of Veterinary Preventative Medicine, College of Veterinary Medicine, The Ohio State University, Columbus, OH 43210, USA; 3 Department of Biology, Baylor University, Waco, TX 76798, USA; katelyn_mckindles@baylor.edu; 4 Center for Reservoir and Aquatic Systems Research, Baylor University, Waco, TX 76798, USA; 5 Great Lakes Center for Fresh Waters and Human Health, Bowling Green State University, Bowling Green, OH 43403, USA; 6 Department of Food Science and Technology, The Ohio State University, Columbus, OH 43210, USA; 7 Infectious Diseases Institute, The Ohio State University, Columbus, OH 43210, USA

**Keywords:** *Planktothrix agardhii*, cyanophages, microcosm, bioremediation, water treatment residuals, lake erie

## Abstract

Cyanobacterial blooms are increasingly becoming more intense and frequent, posing a public health threat globally. Drinking water treatment plants that rely on algal bloom-affected waters may create waste (water treatment residuals, WTRs) that concentrates contaminants. Source waters may contain harmful cyanobacteria, cyanophages (bacteriophages that infect cyanobacteria), and bacteria. Cyanophages are known to affect bloom formation and growth dynamics, so there is a need to understand viral-host dynamics between phage and bacteria in these ecosystems for managing cyanobacteria. This study isolated and characterized lytic cyanophages from WTRs of a HAB-affected lake in Ohio that infect toxic bloom-forming filamentous cyanobacteria *Planktothrix agardhii*. Phage infections in the Lake Erie cyanobacteria culture were examined visually and via microscopy and fluorometry. Whole genome sequencing and metagenomic analyses were also conducted. Observed changes in *Planktothrix* included sheared and shriveled filaments, reduced clumping, and buoyancy changes. Photosynthetic pigmentation was unexpectedly more apparent during phage infection. Metagenomic analyses identified nineteen phages and seven other co-existing bacterial genera. Annotated bacterial genomes contained metabolic pathways that may influence phage infection efficiency. Viral genomes were successfully tied to microbial hosts, and annotations identified important viral infection proteins. This study examines cyanobacterial-phage interactions that may have potential for bioremedial applications.

## 1. Introduction

Cyanobacteria can be found in many freshwater and marine ecosystems around the world, and have increased in frequency, intensity, and geographic distribution [1]. Lake Erie, one of the five Great Lakes in the United States, is one of the most commonly studied and monitored water bodies in the area because of the prevalent and recurring harmful algal blooms (HABs) dominated by the cyanobacteria genus *Microcystis* [2,3,4,5]. Another common recurring genus of cyanobacteria, especially in Sandusky Bay of Lake Erie and in other inland lakes in Ohio, is the toxic bloom-forming filamentous cyanobacteria *Planktothrix agardhii* [6,7]. Some cyanobacterial strains produce cyanotoxins, natural toxins found exclusively in surface freshwater and brackish water, that are harmful to tissues and cells of various organisms [8]. Microcystins (MCs) are the most ubiquitous and well-studied class of cyanotoxins, with microcystin-LR being the most toxic and common of over 100 different congeners [9,10]. MC toxicity and carcinogenicity in both humans and animals, a well-studied topic, is caused by toxin uptake in functional liver cells (hepatocytes) that inhibit protein phosphatases, causing cell alterations that lead to liver damage [11,12,13,14,15].

Freshwater, such as that of Lake Erie, provides adequate drinking water to millions worldwide, serving as one of our primary drinking water sources [16,17]. Drinking water treatment plants (DWTPs) provide safe, clean drinking water while also appropriately disposing of the many waste by-products produced during treatment. One of these by-products, water treatment residuals (WTRs) are a concentrate of all the suspended solids, toxins, and other chemicals collected during treatment [18]. WTRs are typically left in lagoons or other waste disposal areas [18]. As DWTPs produce increasing amounts of WTRs, normal disposal options are becoming more difficult to sustain both environmentally and economically. Because of this, agricultural land application has become an attractive new option for WTRs [19,20,21]. DWTPs treating HAB-impacted water may produce WTRs with concentrated cyanobacteria and associated toxins, causing concern if applied directly to lands growing crops intended for human consumption [22].

Cyanobacterial populations in natural marine and freshwater environments are successfully selected for and regulated by cyanophages, viruses that exclusively infect cyanobacteria [23,24]. Viruses, including cyanophages, are the most abundant organisms on the planet [25,26]. Due to numerous toxin-related public health concerns, the desire is mounting to control cyanobacteria in both water and other matrices, like WTRs. Using cyanophage as a natural biocontrol for cyanobacteria regulation has the potential to be effective for reducing cyanobacteria cell counts for a few reasons [27,28]. Firstly, the ability of viruses to attack specific hosts can prevent entire microbial community collapse when treating blooms. Secondly, algaecide applications typically require monitoring of side effects of treatment, which would not be necessary for cyanophage application.

Although a potential biocontrol, freshwater cyanophage are largely understudied due to the complexity of isolation. Cyanophage that have been isolated and characterized are morphologically diverse and infect numerous hosts [29]. Many freshwater cyanophages have been identified and isolated around the world, but only a handful of them have been successfully sequenced [30,31,32,33]. Viral metagenomics can be challenging due to the relatively limited reference cyanophage genomes, specifically for lesser studied cyanobacteria like *Planktothrix* [31,34,35,36,37,38,39]. Viral genomics is important for understanding viral diversity, co-evolutionary features between viruses and their hosts, and the ways microbial community structures change in response to environmental fluctuations [40]. Based on few viral metagenomic studies in the region, it is confirmed that the lower Great Lakes, including Lake Erie, are abundant with viruses [41]. However, much is still unknown about viral interactions with the entire microbial community in Lake Erie.

While it is important to characterize and analyze phage infection prior to environmental applications, it is also critical to understand the other bacteria in the microbial community, as they may impact cyanobacterial growth and metabolism. Some metagenomic studies have identified and characterized various microbial communities within bloom-affected waters, particularly in Lake Erie, but have focused almost exclusively on bloom communities consisting primarily of *Microcystis aeruginosa* [42,43]. Unlike *Microcystis*, very little is known about *Planktothrix*-dominant microbial communities, particularly those in Lake Erie’s Sandusky Bay and other inland lakes in Ohio.

Therefore, the main objectives of this study were to: (1) morphologically and phenotypically characterize lytic cyanophages isolated from drinking WTRs from HAB-affected regions that specifically target the filamentous cyanobacteria *Planktothrix agardhii*, (2) examine host-cyanophage interactions using various microscopy methods (optical and scanning electron microscopy) and fluorometry measurements of phycocyanin and chlorophyll-a, and (3) conduct a metagenome analysis of the microbial community present in this microcosm. To the best of our knowledge, this is the first study to isolate cyanophages from HAB-affected drinking water treatment residuals, and to isolate phages that specifically target *Planktothrix agardhii* from WTRs. This study not only provides valuable insights into cyanobacteria-cyanophage interactions within a unique microcosm, but it also introduces the potential for bioremediation applications of cyanophage in bloom-affected waters or other matrices. This potential for reducing HAB-producing cyanobacteria and cyanotoxins may be a future practical and novel approach for targeted applications, all for protecting public health and the environment.

## 2. Results

### 2.1. Optical Observation and Fluorometry

**Figure 1:**
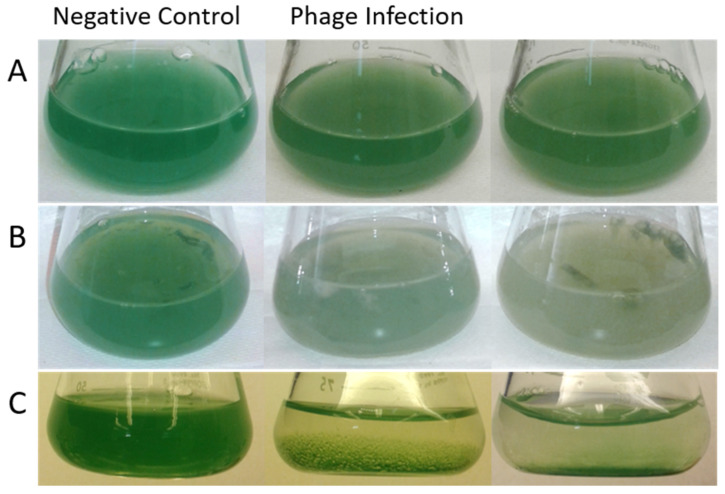
Culture of cyanobacteria *Planktothrix agardhii* without (negative control) and with phage infection, two photos for observation. (**A**) Early stage infections, 2 days. (**B**) Late stage infection, 14 days. (**C**) Early stage infection, 2 days, exhibiting a unique phenotype.

Monitored cultures exhibited various phenotypes caused by cyanophage infection, some previously uncharacterized (Figure 1). During initial phage infection stages (2 days; hereafter described as early stage infection), host cyanobacteria began to exhibit changes in pigmentation, from a bright blue-green to a yellow-green color (Figure 1A). Cyanophage-infection cultures also started to exhibit a loss of filament clumping compared to the negative control culture. Following a two-week incubation period (14 days; hereafter described as late stage infection) of cyanophage, the host filaments lost much of their color, with filaments changing to white, as cell damage can result in pigmentation loss (Figure 1B) [44]. Clumps that managed to stay intact started changing to yellow-green. One sample culture (Figure 1C) exhibited a different phenotype from both the early and late stage cyanophage infections (Figure 1A and 1B, respectively), where the cyanobacteria layered the bottom of the flask and clearing the majority of the culture. The control cyanobacteria remained a healthy culture, the solution uniform and filaments a deep blue-green.

**Figure 2:**
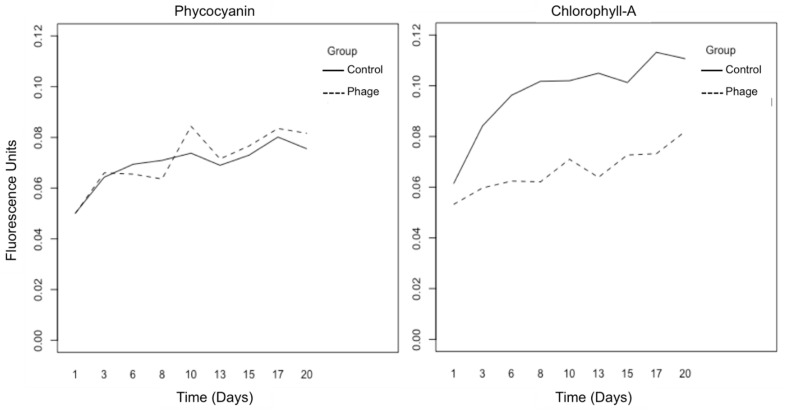
Cyanobacteria changes in growth, measured as phycocyanin (**left**) and chlorophyll-a (**right**) fluorescence units over time (in days). Negative control cultures are the solid lines and phage-inoculated cultures are the dashed lines.

Fluorometry tests for the phage-inoculated cultures showed unexpected trends in growth biomass, measured in fluorometry units, when compared to the control cultures through the two-week inoculation period (Figure 2). For phycocyanin, both the control and the phage-inoculated culture significantly increased in pigmentation over time (*p*-value < 0.001) but no significance was observed between trend lines for the control and phage-inoculated cultures (*p*-value > 0.05). However, there was a significant difference between control and phage-inoculated cultures for chlorophyll-a pigmentation levels, the former exhibiting a significantly higher fluorescence trend level compared to the latter over time (*p* < 0.001).

### 2.2. Optical and Scanning Electron Microscopy

**Figure 3:**
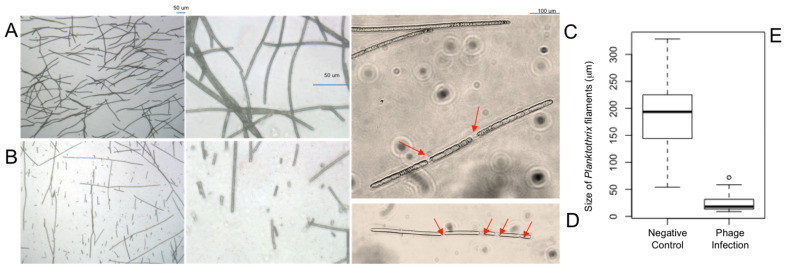
Optical light microscope imagery of the cyanobacteria *Planktothrix agardhii* (**A**) without and (**B**) with phage during late-stage infections, exhibited with two different total magnifications of 250× (**left**) and 1000× (**right**), respectively. (**C**,**D**) Closer look at filament fragments during early stage infection cycles at 1200× total magnification. Red arrows indicate noticeable breaks in a single filament. (**E**) Box plot of the size of filaments counted for both the negative control sample and the phage infection sample.

Optical microscope observation results showed a clear distinction between control and phage-infection cultures (Figure 3). In the control cultures (Figure 3A), the filaments were long, full-sized, filled-out, and crowded due to exponential growth, with a mean filament length of 190.4 μm (SD = 67.3, sample number = 50). When examining the initial progression of the early stages of infection (2 days), single long filaments were beginning to be sheared into numerous fragments due to simultaneous infection but were not yet scattered in the culture (Figure 3C,D). In the late stage phage-infected cultures (Figure 3B), the filaments exhibited a mostly sheared phenotype, reduced to small, scattered pieces, with a mean filament length of 24.12 μm (SD = 15.4, sample number = 50). Filament sizes in the control cultures were significantly different to the filament sizes in the late stage phage-infected cultures (*p*-value = 2.2 × 10^−16^; confidence interval: 146.8–185.9) when tested using both a normal (*t*-test) and non-normal (Wilcoxon) significance test. These significant differences are also exhibited in a box-and-whisker plot, showing much larger filaments in the negative control vs. the phage-infection cultures (Figure 3E). Phage inoculations through several serial dilutions exhibited the same symptoms and changes in culture.

**Figure 4:**
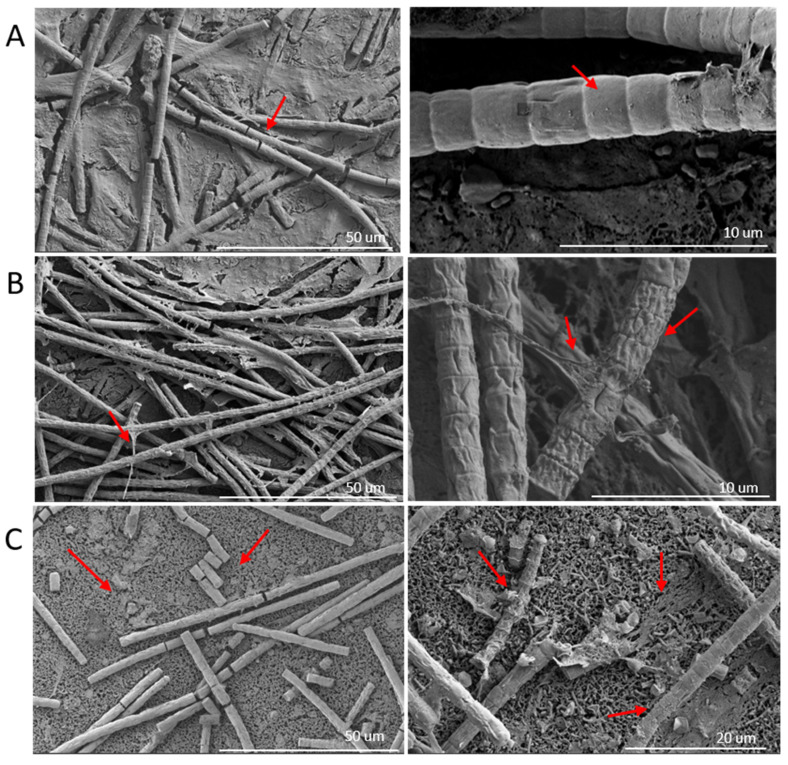
Scanning electron microscope imagery of the cyanobacteria *Planktothrix agardhii.* (**A**) Negative control without phage at 2000× and 10,000× total magnification. (**B**) With phage during early-stage infections at 2000× and 10,000× total magnification. (**C**) With phage during late-stage infection at 2000× and 8000× total magnification. (**A**–**C**) Red arrows indicate points of discussion, outlined in the Results.

SEM images revealed notable, previously undocumented changes in *P. agardhii* during phage infection, with red arrows indicating particular points of interest (Figure 4). The negative control (Figure 4A) again showed long, healthy filaments. At 2000× magnification, some breaks were seen in the filaments, due to the nature of sample preparation for SEM (44). At 10,000× magnification, the filaments looked healthy, with plump single cells that make up each filament, as well as some heterogeneous bacteria on and below the cells. The phage-infected culture (Figure 4B,C) showed the filaments in two drastically different states. The cyanobacteria from the early phage-inoculated cultures are shown with long, intact filaments (Figure 4B). However, the individual filaments not only appear shriveled, but large amounts of extracellular matter appear to be coming from or on top of the filaments. A late phage-inoculated sample exhibited what appeared to be a more progressed phage-infection, with many of the filaments already sheared and scattered across the field, a phenotype consistent with what was observed in the optical light microscope images (Figure 4C). The field background also contains large amounts of heterogeneous bacteria, growing on and around the sheared filaments. In both stages of phage infection, the filaments appear stressed or in cell death.

### 2.3. Next Generation Sequencing

#### 2.3.1. Metagenome-Assembled Genomes

**Figure 5:**
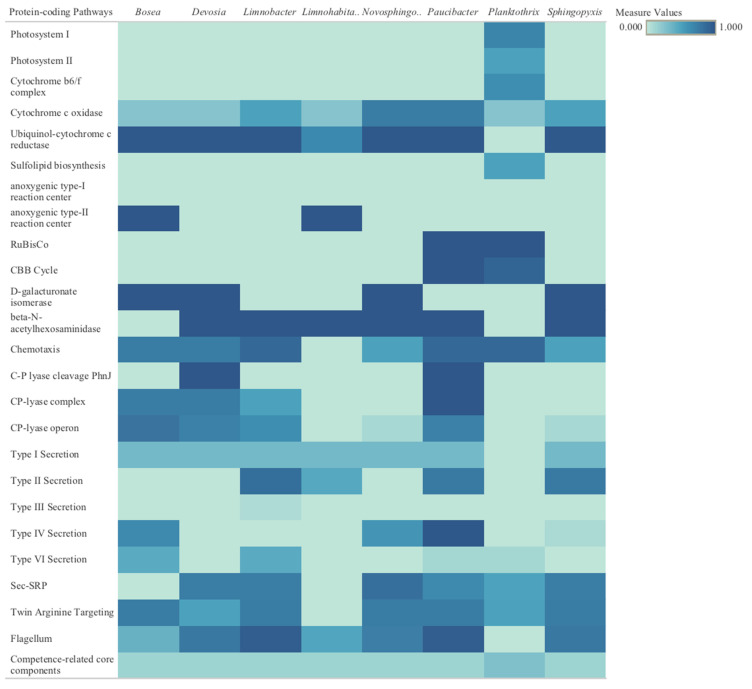
Metabolic pathways for eight identified bacterial genomes in this microcosm. Bacteria in order from left to right: *Bosea*, *Devosia*, *Limnobacter*, *Limnohabitans*, *Novosphingobium*, *Paucibacter*, *Planktothrix*, and *Sphingopyxis*, broken down by protein-coding pathways. Dark colored blocks are associated with the full presence of genes associated with a pathway (full pathway completion ranked as 1.0), while light colored blocks are associated with no presence of significant genes in the pathway (ranked as 0.0).

Next-generation sequencing and genome-resolved metagenomics recovered eight medium-to-high quality metagenome-assembled genomes (MAGs) present in the cyanobacteria cultures. The following MAGs were identified down to either the genus or species level: the cyanobacteria *Planktothrix agardhii*, as well as *Devosia* sp001556025, *Bosea* sp., *Paucibacter* sp., *Novosphingobium* sp., *Sphingopyxis* sp., *Limnohabitans* sp., and *Limnobacter* sp002954425. Metabolic pathways for the eight identified bacterial genomes were defined (the full list of predicted protein-coding pathways is provided in the Supplementary Materials, as well as Appendix A). Extrapolated metabolic pathways of most interest, for both *P. agardhii* and the other microbial genomes, represented an array of expected and unexpected carried genes relevant to both general survival and survival through external stress, such as phage infection (Figure 5). *P. agardhii* carried genes important for photosynthesis and energy conversion, as well as sulfur uptake for cell stabilization during photosynthesis. Enzymes associated with major carbon fixation cycles were also present in the cyanobacteria genome. Additional details regarding pathways and functions present and absent in the various genomes are discussed in more detail in Appendix B.

#### 2.3.2. Viral Genomic Fragments and Phage–Host Associations

**Figure 6:**
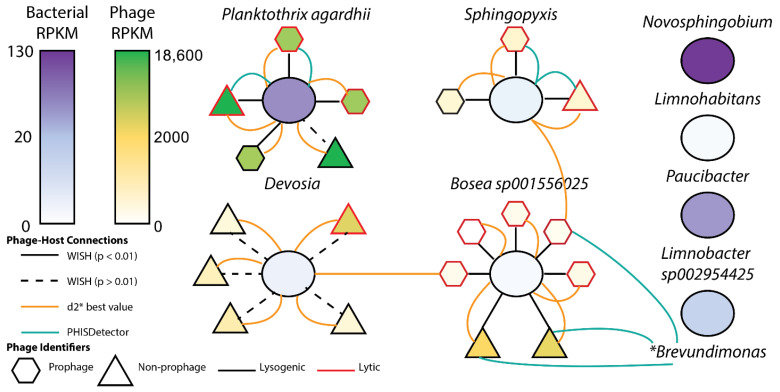
Defined phage-host interactions using four methods: WIsH (*p* < 0.01 and *p* > 0.01), VirHostMatcher d2* best value, and PHISDetector. The method used was distinguished by the colored connections between shapes. RPKM distinguishes the completeness of the genome, with darker colors indicating more completeness. Circles indicate bacterial genomes. Triangles and hexagons are phage, and were identified as prophage, non-prophage, lysogenic, and lytic.

Nineteen unique bacteriophage genomes in the culture samples were identified, the majority of which were low quality, with one medium quality, and one high quality, complete circular genome. Since these viral sequences are not assumed to be whole viral genomes, they are characterized here as viral genomic fragments (VGFs) [45]. Phage–host interactions were defined using four distinct methods and revealed significant links between fourteen VGFs and four host microbial MAGs (Figure 6). Ten of the 19 VGFs were identified as prophage and were linked to *Planktothrix agardhii* (three genome fragments), *Sphingopyxis* sp. (two genome fragments), and *Bosea* sp001556025 (five genome fragments). In total, four VGFs (three prophage and one non-prophage) had significant links to *Planktothrix agardhii*, predicted by WIsH (*p* < 0.01), VirHostMatcher, and PHISDetector. One viral genome had *P. agardhii* as a potential host, but this link was not statistically significant. The lifestyles of the viral genomes linked to *P. agardhii* were predicted (lytic or lysogenic), revealing three lytic and two lysogenic phage genomes.

Three VGFs had significant associations with *Sphingopyxis* sp., two lytic and one lysogenic phage genome. One phage genome was predicted by VirHostMatcher (d2* score) to be associated with *Sphingopyxis*, yet the WIsH methodology predicted a significant association with *Bosea* sp001556025. Six phage genomes were associated with *Devosia* sp.; however, none of these were statistically significant connections, and one of these phage genomes was predicted by WIsH to be associated with *Bosea* sp001556025. Five prophage genomes and two phage genomes were significantly associated with *Bosea* sp001556025. Interestingly, all prophages were predicted to be lytic, while the phage genomes were predicted to be lysogenic. The two lysogenic phages associated with *Bosea* were predicted by PHISDetector to be associated with *Brevundimonas* sp., which was not a recovered genome in this sample. However, using singleM to reconstruct single-copy marker genes to infer taxonomy, we confirmed the presence of *Brevundimonas* in this microcosm. Further links were identified between one prophage associated with both *Sphingopyxis* and *Bosea*, and *Brevundimonas*, using PHISDetector. No viral genomes were associated with *Novosphingobium*, *Limnohabitans*, *Paucibacter*, or *Limnobacter* sp002954425.

#### 2.3.3. Phage Genetic Analysis

The identified phage contigs contained a range of genes, anywhere between 6 and 90, with a mean gene count of 18. Within the VGFs, 29% of the protein-coding regions shared significant similarity to known sequences within the BLAST database (version 2.2.26), indicating that 71% of genes had no known match to the reference database. Identified genes were categorized into three groups: genomic proteins, structural proteins, or phage-specific proteins. Nearly half of all identified genes were genomic proteins, while the other half were split between structural and phage-specific proteins.

**Figure 7:**
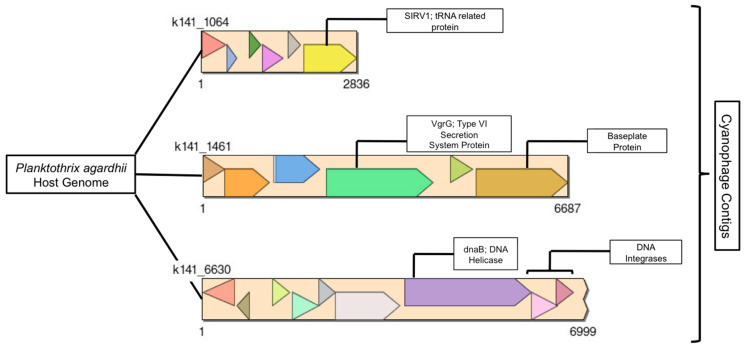
Three cyanophage contigs (k141_1064, k141_1461, and k141_6630) were found within the *Planktothrix agardhii* host genome as putative prophage, with several identifiable genes. Contigs were annotated and visualized using BLAST and SimpleSynteny, respectively.

As mentioned above, three phage contigs were found within the *Planktothrix* genome as putative prophage and contained several genes that were identifiable (Figure 7). These three contigs were short, with few genes encoding limited known proteins. The three contigs contained six known annotated proteins from either the genomic, structural, or phage-specific protein groups. Genomic proteins included a DNA helicase and a tRNA protein. Structural proteins included a type VI secretion system-like protein and a baseplate protein. Defined phage-specific proteins were DNA integrases. The remaining bacteriophage contigs were a mixture of lysogenic and lytic, with some found within other MAGS, such as the *Bosea* sp001556025 species and the *Sphingopyxis* species. A high-quality, complete circular genome was identified, containing 90 genes, 28% of which are protein-coding (Appendix C).

## 3. Discussion

*Planktothrix agardhii* are filamentous toxic bloom-forming cyanobacteria, and are highly prevalent in Sandusky Bay, Lake Erie, as well as many inland lakes in Ohio and elsewhere [46,47,48,49]. Lake Erie, as well as many inland lakes, act as recreational water and drinking water sources for much of Ohio. Many of the Ohio water bodies have repeated annual blooms, which feed into many DWTPs across the state [50]. WTRs collected in 2018 from two Ohio DWTPs were screened for lytic cyanophages against *P. agardhii*. The methods described for viral isolation from WTR samples were adapted from soil isolation protocols and can be used for further study of WTR microbiomes and viromes [35]. These methods are ideal for non-specialized research labs looking to investigate environmental systems but may have financial and expertise limitations.

### 3.1. Optical Observation and Fluorometry

The observed visual changes in the cyanobacteria (Figure 1) may be the most effective and most appropriate way to visualize a phage infection for *P. agardhii* due to the drastic changes in culture. The changes in both clumping and color are due to the initial shearing of the *Planktothrix* filaments followed by cell death, phenotypes that have been characterized by other *P. agardhii* phage infections [34,51]. The phenotype exhibited in Figure 1C is unique, as the cyanobacteria clear the suspension and exclusively line the bottom of the flask. There are at least two possible explanations. Firstly, cyanobacteria have many gas vesicles that allow them to stay afloat in various water bodies, which act as a crucial mechanism for buoyancy [52,53]. It is possible that phage infection could have caused perforation of the cell wall or intracellular vacuoles, forcing the cyanobacteria to sink to the bottom of the flask. Another possibility could be that the phage infection caused cyanobacterial stress, yielding the formation of a biofilm-like structure. Bacteria commonly form biofilms in order to protect from outside stress, invasion, or disturbances of some kind, such as scum- or mat-like biofilms that protect cyanobacteria from viral infection [54,55]. For both instances, stress induced by phage presence caused this interesting phenotype not previously visualized and reported in *P. agardhii*.

For *M. aeruginosa*, the most commonly studied toxic bloom-forming cyanobacteria genus, the use of the photosynthetic pigments chlorophyll-a and phycocyanin is an effective monitoring technique for blooms in lakes and other affected water bodies [56]. However, using these same pigments for monitoring of *Planktothrix* (Figure 2) may not be as effective for comparing healthy and phage-infected cultures, even though statistical significance was observed between control and phage cultures for chlorophyll-a. Optical microscopy (Figure 3) showed the shearing of cyanobacteria filaments during phage infection, and the culture became more uniform with smaller, more distributed blue-green cells. Statistical tests, along with the graph in Figure 3E, show a significant difference in filament length between our negative control and phage-infected cultures, exhibiting a strong morphological change in *Planktothrix* cells. In nature, *P. agardhii* blooms are not bright blue-green, as they are naturally less brightly pigmented than their competitor *M. aeruginosa* [48]. Moreover, in their natural state, *Planktothrix* can form clumps (not to be confused with scums) or biofilm-like structures on a water surface during bloom formation [57]. Especially in culture, this quality makes it difficult to sample a uniform solution of host cells or attempt to homogenize the culture without disturbing the community. Because the phage infection causes a more uniform distribution of pigmentation due to the shearing of filaments, unlike its natural control phenotype with more uneven and clumped cell groups, it is possible that fluorometry exhibits results that differ from what is seen for other types of cyanobacteria phage infections, and may explain the results in Figure 2.

### 3.2. Optical and Scanning Electron Microscopy

The shearing of the long filaments (observed in Figure 3) is a phage-infection phenotype that has been previously reported for a *P. agardhii*-infecting phage PaV-LD (34). Additionally, the average filament size of *P. agardhii* for both the control and the induced phage infection is similar to that of the same study. SEM imaging of *Planktothrix* filaments exhibited a clear phenotypic difference between the control and the phage-infected solutions (Figure 4), indicative of cell stress. To the best of the author’s knowledge, SEM images of *Planktothrix* during phage infection have not been previously published, and the observed qualitative results have not been previously reported.

### 3.3. Next Generation Sequencing

#### 3.3.1. Metagenome-Assembled Genomes

The next-generation sequencing and genome-resolved metagenomics successfully reconstructed eight unique bacterial genomes in this community, alongside *Planktothrix agardhii*. Excluding *Planktothrix*, these bacteria are Gram-negative, rod-shaped, motile, oxidative bacteria that have been previously isolated from a range of environments. *Devosia*, *Novosphingobium*, *Limnobacter*, *Limnohabitans*, and *Bosea* species have all been isolated from a range of freshwater-associated environments, such as soils, beach and lake sediments, various surface waters, and wastewater treatment plants around the world [58,59,60,61,62,63,64,65]. *Paucibacter* and *Sphingopyxis*, two genera of bacteria isolated from freshwater lakes and lake sediments, are found to degrade hepatotoxins like MCs [66,67,68,69,70]. The reconstructed genomes of *Paucibacter* and *Sphingopyxis* were analyzed to identify MC-degrading genes, but no homologs of those previously characterized bacteria were found. However, this may be the result of under-characterized genes for MC-degradation pathways in this region rather than the absence of these genes, as has been previously shown [71]. Further isolation and genomic analyses would be required to best understand these MC-degradation pathways.

Some of the bacteria identified here have also been studied in other cyanobacterial bloom communities. Two large-scale studies investigated the microbial communities in bloom-infected waters and identified *Bosea*, *Limnobacter*, *Alphaproteobacteria*, *Novosphingobium*, and *Sphingopyxis* [42,43]. Of these bacteria, *Bosea* and *Novosphingobium* enhanced the growth of *Microcystis* in the community, while *Sphingopyxis* did not affect cyanobacteria growth. These bacteria may play similar roles in this Lake Erie microcosm, especially in a rather unstudied bloom community (one dominated by *Planktothrix*), but again, further studies would be necessary to characterize microbial metabolisms and confirm this hypothesis.

The recovered microbial genomes were annotated (Figure 5) and contained many important proteins associated with major bacterial cell function or survival at similar abundances to one previous metagenomic study of bloom-affected waters [72]. Examining the many metabolic pathways of these bacteria allows for the improved understanding of some of their survival mechanisms within this microcosm, while also revealing the interactions with other community members in the same ecosystem, especially under changing environmental circumstances. Understanding microbial metabolisms in a community reveals potential co-metabolisms and environmental adaptations, and these interactions are crucial for developing biocontrols to regulate or control microbial community structure and dynamics. One example pathway that was identified and considered important for many bacteria is competency, the ability for cells to actively take up foreign environmental DNA. Competency is an important contributor to horizontal gene transfer, while also allowing for the uptake of viral DNA or plasmids and influencing the ease with which viral infection may occur [73]. This easily enables viral DNA movement across cell membranes and may have implications on phage infectivity and integration into host genomes [74]. *Planktothrix* specifically was missing some proteins that have been previously identified, such as those for nitrogen fixation, biofilm formation, and cyanotoxin production [75,76,77]. *P. agardhii* had a 90% complete genome, and, thus, the missing genes may be a result of incomplete sequencing and genome reconstruction rather than the actual absence of those genes. However, further study is required to confirm this hypothesis. Additional pathways of interest for *Planktothrix* and the other bacteria are described in detail in Appendix B. For future studies, this approach can serve as a screening tool to identify some of the key organisms in the community, but it would benefit from additional screening and isolation of individual species to conduct clearer sequencing.

#### 3.3.2. Viral Genomic Fragments and Phage–Host Associations

Nearly twenty unique and diverse phage genomes were successfully identified following methods that, to the author’s knowledge, have not been used for WTR sample isolation. Although some studies have investigated phage genomes in marine and freshwater environments, much is still unknown. Viral gene databases represent only a small glimpse of the genes and proteins present in these vastly diverse organisms [31]. Comparison of the phage genomes to previously sequenced genomes, especially those in freshwater systems, revealed the lack of characterization of many viral genes (e.g., those defined as hypothetical). With over 70 percent of the genomes undefined, this leaves extensive room for future analysis, and the possibility for new discoveries of phages in WTRs. As methods improve over time, it becomes more likely that hypothetical genes are defined. Moreover, phage genome studies are needed to improve the databases that provide such annotations and enable better understanding of these important microorganisms for the future.

Interactions between microbial and phage genomes were identified and revealed viral-host links between four microbial genomes and the nineteen phage genomes (Figure 6). Phage genomes linked to *P. agardhii* were exclusive to that host, whereas the other microbial genomes (*Devosia*, *Bosea*, *Sphingopyxis*) had phage genomes tied to multiple hosts. This is likely indicative of the general exclusivity of cyanophage to cyanobacterial hosts, whereas bacteriophage genomes tend to have a more broad distribution of hosts [31,78]. More likely, though, this may be the result of different methodologies used to tie viruses to hosts. Because some methods used reference databases and some did not, phage linked to multiple hosts from different orders (all were members of *Alphaproteobacteria* class) may be false positives [79]. However, previous studies have identified phage capable of infecting hosts from different orders, so this finding may be possible [80]. Some phage genomes may have unidentified links to other microbial community members that did not assemble or bin well, such as *Brevundimonas* sp. *Brevundimonas* has been identified in freshwater lakes and bloom-infected waters, influencing cyanobacteria communities in various ways [42,81,82]. Expanding this study to include replicates, and performing deeper sequencing may reveal additional microbial bins and phage–host links.

Several phage genomes linked to *P. agardhii* were characterized as lytic, revealing a promising biocontrol target for toxin-producing cyanobacteria. In addition, the phage linked to *P. agardhii* had the highest RPKM compared to all other phage genomes, indicating the high abundance of this phage in this microcosm. The presence of *Paucibacter* and *Sphingopyxis* in the microcosm, previously identified as MC-degraders, may indicate a community metabolism involving MC production (*P. agardhii*) and degradation (*Paucibacter*, *Sphingopyxis*). The combined finding of these putative MC-degraders and lytic phage linked to toxic bloom-forming *Planktothrix* may lead to a two stage, targeted biocontrol during bloom seasons, to both remove toxin and reduce cells capable of producing toxin. However, further research is needed to confirm ability of these bacteria to degrade MC or other toxins, and to discern the optimal environmental conditions for phage lysis of host cells within the environment.

#### 3.3.3. Phage Genetic Analysis

Three prophage genomes associated with the *P. agardhii* host genome contained genes annotated as genomic, structural, and phage specific proteins (Figure 7). Genomic proteins relate to DNA replication, binding, folding and packaging, such as DNA polymerases and helicases, enzymes, and transfer RNAs (tRNAs). These proteins are crucial for effective viral replication within host cells, allowing for rapid infection and proliferation within a system. Both DNA helicase and tRNAs were present in these contigs [83,84]. These contigs were compared to PaV-LD and other Sandusky Bay phage sequences [36,51]. While the PaV-LD genome, Sandusky Bay cyanophage sequences, and the experimental contigs contain genes for a DNA helicase, integrases, and tRNA proteins, the experimental contigs also contain genes for a baseplate and a Type VI secretion system, which are distinctly absent from the other genomes. This is likely due to the incompleteness of these cyanophage contig fragments, and additional DNA extraction and metagenomics would be necessary to better understand these *Planktothrix* cyanophage. Additional explanation for the identified proteins is described further in Appendix D.

## 4. Conclusions

This study successfully extracted cyanophage from WTR samples from two DWTPs, visualized cyanobacteria phage infection, extracted DNA, reconstructed and characterized eight microbial genomes from a metagenomic assembly, recovered nineteen phage genomes, and tied phages to hosts within this small microcosm. This metagenomic approach enabled analysis of the system without conducting time-consuming isolation experiments for each of the microorganisms, which would be especially difficult from complex environmental matrices such as WTRs. These methods can potentially be applied in other labs while providing the same results, particularly for less technical labs. The inexpensive procedures and relatively quick analyses make this ideal for microcosm analyses in the future. The identification of the present microbial community provides insight on the possible community dynamics and interactions that are at play in this system, but more studies are necessary to fully understand this microbiome.

Moreover, the cyanophage identification, along with the host-phage culture experiments, provided valuable insight into this complex relationship. These phage-cyanobacteria interactions are especially insightful when cyanobacteria and their toxins become a public health concern. When toxin-containing cyanobacteria are lysed, cyanotoxins are expected to be released into the surrounding waters [35]. This release of cyanotoxins can overwhelm drinking water treatment plants and can lead to instances of breakthrough and cause potentially serious health crises, like what occurred in Toledo, Ohio in 2014 [85,86]. While control of cyanobacteria manages one challenge for water bodies, the release of cyanotoxins causes another. For cyanophage to be a viable and effective biocontrol, strategic action would be necessary to ensure cyanophage treatment also addressed the released cyanotoxins [87,88,89].

Future studies could examine sequential applications of cyanophage on cyanobacteria followed by cyanotoxin quantification, to understand toxin release during applications. Future studies could also examine conditions in which phage interact more or less, such as with what has already been examined with UV exposure during phage infection on *Microcystis* [90]. Additionally, with this initial screening of this microbiome, isolation of the various microbes to perform more in-depth sequencing would be beneficial. A holistic approach to successful biocontrol that incorporates phage infection could be a non-toxic or non-invasive strategy for managing nuisance cyanobacteria in small water bodies, as well as for application in other HAB-affected matrices like WTRs that may be used in future downstream applications. Further studies of phage-cyanobacteria interactions in various matrices, along with cyanotoxin release, in HAB-affected areas are necessary prior to applications of phage as an aquatic biocontrol for the future.

## 5. Materials and Methods

### 5.1. Cyanobacteria Host Maintenance and Monitoring

*Planktothrix agardhii* was isolated from western Lake Erie water samples from a previously unpublished study, and grown in CT media at 24 °C under 12 h light cycles at 2-week intervals [1,91]. Each subsequent experiment used fresh cyanobacteria culture grown for 2 weeks in fresh CT media, grown to an approximate concentration of 1 × 10^7^ cells/mL. As a proxy for cyanobacterial growth, chlorophyll-a and phycocyanin pigment levels were monitored using an AquaFluor Handheld Fluorometer (8000-010 Turner Designs, Sunnyvale, CA, USA), at excitation wavelengths of 460 ± 20 nm and 595 ± 20 nm, respectively [56]. Calibration of the fluorometer was conducted following manufacturer instructions (https://docs.turnerdesigns.com/t2/doc/tech-notes/S-0242.pdf [accessed on 7 December 2021]).

### 5.2. Cyanophage Extraction and Elution from WTR Samples

WTR samples were collected from two drinking water treatment plants (DWTPs) in western Ohio in 2018 [92]. Viral particles were extracted and eluted from these samples using the methods described by Wommack et al. [93] Unsure if wet or dry WTRs would be appropriate for screening, both a wet sample and a dry sample were extracted and tested. In short, 5.0 g of WTR was vortexed with 1% potassium citrate extraction buffer (15 mL, pH 7.0), placed on ice for 30 min, then sonicated at 0 °C (five-one minute cycles with 30 s rest and brief mixing) using a ¾ gallon benchtop sonicator (80 watts, 40 kHz, model 2510, Branson Ultrasonics, Danbury, CT, USA). The sample was then centrifuged at 3000× *g* for 30 min at 4 °C (Thermo Scientific CL2 Low Speed Table Top Centrifuge, 175 watts, Grand Island, NY, USA). The supernatant was filtered through a sterile 0.22 μm pore size paper filter (CAT#SLGP033RS Millipore Corp., St. Louis, MO, USA). The final eluent was used for further analyses.

### 5.3. Cyanophage Inoculation and Screening

Determining viral activity using plaque assays for filamentous cyanobacteria, like *P. agardhii*, is insufficient due to their motility [29]. Therefore, the following methods modified from Gao et al. [34] and Wilhelm et al. [94] allowed for viral screening of lytic activity. Briefly, 200 μL of the extracted viral concentrate was inoculated in triplicate into 1 mL of exponentially growing *P. agardhii* host culture using 24-well plates, mixed, and incubated at 24 °C under the same light conditions as described previously. Negative control wells were inoculated with 200 μL autoclaved PCR-grade water (CAS#7732-18-5, Thermo Fisher Scientific, Waltham, MA, USA). Three experimental replicates were completed for screening. Plates were monitored daily for lytic activity via chlorophyll-a and phycocyanin measurements coupled with light microscopy observations (Zeiss Axioskop 5, Carl Zeiss Microscopy LLC, White Plains, NY, USA) through a 7-day incubation period.

Samples were compared between the wet and dried WTR-extracted viral particles. The wet WTR proved to show increased lytic activity, so the wet WTR samples were used and discussed hereafter. Once lytic activity was confirmed by the clearing of solution, visible cell death and/or changes in fluorometry measurements, samples were serially diluted (six cycles) and further screened. To propagate infectious phage, 1 mL of the final dilution was centrifuged to separate cell debris and the supernatant was inoculated into *P. agardhii* host culture at a ratio of 1:50 by volume. Replicates were left to propagate over 2 weeks, each to a final culture volume of 250 mL.

### 5.4. Cyanophage Collection and Concentration

Select phage samples were collected and isolated using the cation-coated filter methods from Haramoto et al. [95], and adapted similarly to Jiang et al. [90]. Briefly, 250 mL cyanobacterial-cyanophage cultures were pre-filtered (vacuum) through 20 μm pore size nylon filters (47 mm diameter, GVS Magna^TM^ Membrane Filters, Fisher Scientific, Waltham, MA, USA) to remove host cells. The filtrate was then passed through 0.45 μm Whatmann white gridded filters (HAWG04756 EMD Millipore Filter, MilliporeSigma, St. Louis, MO, USA) coated with AlCl_3_ (5 mL, 250 mM). The filter was rinsed with H_2_SO_4_ (200 mL, 0.5 mM, pH = 5.0), and the captured phages on the filter were eluted into 10 mL using NaOH (10 mM, pH = 10.8). The samples were neutralized with 100× Tris-EDTA buffer (100 μL) and concentrated to 3 mL using Amicon Ultra-15 Centrifugal Filters (MilliporeSigma, St. Louis, MO, USA) and PBS buffer (1×, 1 mL) via centrifugation at 3000× *g* (24 °C, for 3–5 min).

### 5.5. Cyanophage–Host Interactions In Vitro

Four cyanophage sample concentrates and PCR-grade water (for control) were inoculated into exponentially growing *P. agardhii* host culture at a ratio of 1:100 by volume and incubated for 2 weeks under the previously described growth conditions. Phycocyanin and chlorophyll-a levels were measured to monitor changes in host health during viral infection using a microplate reader (SpectraMax Plus 384 Microplate Reader, Molecular Devices, San Jose, CA, USA). Samples (500 μL) were taken every 24 h throughout the growth period, and stored at −80 °C for further analysis. Each sample was tested in duplicate, and each experiment was run in triplicate. As noted in the manufacturer’s manual, calibration of the microplate reader is automatically conducted before first kinetic read and before every endpoint reading (https://www.egr.msu.edu/~scb-group-web/blog/wp-content/uploads/2012/07/plate-reader1.pdf [accessed on 7 December 2021]).

### 5.6. Optical Light Microscopy

Samples from the phage–host culture In Vitro experiments were collected and prepared for optical light microscopy using dry-fixing techniques following facility protocols (similarly to example protocols, such as via atcc.org/resources/culture-guides/introduction-to-microbiology [accessed on 7 December 2021]). Briefly, 20 μL of sample were placed on a glass slide (Superfrost^TM^ Plus, Thermo Scientific, Grand Island, NY, USA), left to air dry, followed by the addition of a slide slip (Fisherbrand^TM^ Premium Cover Glass, Fisher Scientific, Waltham, MA, USA), and fixed under flame to remove excess moisture and ensure no movement. The slides were examined using a light microscope (Zeiss Axioskop 5, Carl Zeiss Microscopy LLC, White Plains, NY, USA) and digital images were captured with a QICLICK Digital CCD Camera (F-M-12, QImaging, Surrey, BC, Canada). Photos were taken at the following magnifications: 250×, 1000×, and 1200×.

### 5.7. Scanning Electron Microscopy

Based on optical light microscopy, two samples along with a negative control were chosen for scanning electron microscopy (SEM), modified from a protocol developed by the Campus Microscopy and Imaging Facility at The Ohio State University [96]. Briefly, 15 mL samples were centrifuged (8 °C, 7000 rpm, 10 min), the supernatant discarded, and remaining cells were washed in PBS (1×, 1 mL, 3 cycles). The cells were resuspended with PBS (1×, 1 mL) and hand-filtered with 0.22 μm pore size filters (MF-Millipore, CAT# GSWP02500, Bedford, MA, USA) using a pre-sterilized 10 mL syringe. The cyanobacteria on the filters were fixed by immersion in glutaraldehyde (5%) in phosphate buffer (0.1 M, 7.4 pH) and placed in 4 °C for 72 h. The filters were then rinsed with PBS (1×, 2.5 mL, 3 cycles of 5 min) prior to drying.

Filters were dried with increasing ethanol solutions (2.5 mL, 50–100%, 10 min rinses), followed by increasing solutions of ethanol-hexamethyldisilazane (2.5 mL, 0–100%, 15 min rinses, Electron Microscope Sciences, CAT#16700, Hatfield, PA, USA) and then placed in a desiccator (Fisherbrand^TM^ Heavy Glass Nonvacuum, Fisher Scientific, Waltham, MA, USA) for final drying. Samples were mounted onto stubs, coated with gold in a sputter coater unit (PELCO^®^ Model 3 Sputter Coater 91000, Ted Pella Inc., Redding, CA, USA) and examined using an FEI Apreo LoVac High Resolution SEM (1.00–10.00 kV, Thermo Fisher Scientific, Waltham, MA, USA) at the Center for Electron Microscopy and Analysis at The Ohio State University (Columbus, OH, USA). Photos were taken at the following magnifications: 2000×, 8000×, and 10,000×.

### 5.8. DNA Preparation for Whole Genome Sequencing

DNA from the concentrated In Vitro microcosm samples were extracted using a Phage DNA Isolation Kit (Norgen Biotek Corp., Thorold, ON, Canada) following manufacturer instructions. Extracted DNA was concentrated to 15 μL (>20 ng/μL concentration) and sent to CosmosID (Rockville, MD, USA) for sequencing. DNA libraries were prepared using the Thermo Fisher IonXpress Plus Fragment Library Kit, according to manufacturer’s protocols. DNA quantity was confirmed with a Qubit Fluorometer (Thermo Fisher Scientific, Waltham, MA, USA). Four biological replicate libraries were then sequenced using a Thermo Fisher Ion S5 XL sequencer.

### 5.9. Bioinformatics Analysis

Single-end sequence reads, provided after sequencing by CosmosID, from four replicate samples were concatenated then trimmed using Cutadapt [97]. SingleM was used on trimmed single-end reads to identify conserved single copy marker genes and identify taxonomic profiles of the sample (https://github.com/wwood/singlem [accessed and run on 5 November 2020]). Metagenomic reads were assembled using MEGAHIT v.1.1.3 [98] with its default multi-k-mer approach. Viral contigs were identified using VIBRANT [99], which classifies phage contigs as lytic or lysogenic, assesses viral genome quality (low, medium, high), and identifies and annotates protein-encoding genes. For this, default metagenome mode was used with no additional parameter adjustments. Metagenome assembled genomes (MAGs) were reconstructed using two binning programs, MetaBat2 [100] and CONCOCT [101], each using their default settings. For MetaBat2, the minimum contig length was 1500 bp, and for CONCOCT, the minimum was 1000 bp. Default bin size thresholds were applied in both tools. MAGs were refined using metaWRAP’s bin refinement tool [102] and retained if >50% completion and <5% contamination, as assessed by CheckM [103]. While higher completeness thresholds (70–80%) are commonly adopted for functional genome analysis, the current study focused on ecological inference. Therefore, we applied a more inclusive completeness cutoff in order to maximize the recovery of cyanophage and cyanobacteria-associated bins from this complex water treatment waste environment, while maintaining stringent contamination control. Taxonomy was assigned using GTDB-Tk v. 0.3.2 [104]. Genome annotations were performed by translating genome fasta files to amino acid files with prodigal v. 2.6.3 [105], then annotated using KOFAMScan [106]. KEGG pathway coverages were estimated and visualized using KEGGDecoder [107]. A pathway Euclidian distance score was used for the MAGs in order to distinguish differences between completions of each pathway. The pathway differences in each bin were compared and visualized using Tableau Desktop v. 2020.2 (NorthEdge, 1621 N 34th St., Seattle, WA, USA).

Viral-host relationships were assessed using four methods, adapted from a previous study [79]. First, blastp was used to identify which viral contigs were prophage encoded within the reconstructed MAGs, requiring 100% alignment, 1 or less mismatches, ≥97% identity, and e value ≤ 1.00 × 10^−10^ [108]. Second, VirHostMatcher with d2* dissimilarity scores compared viral contigs to MAGs in the sample, taking the lowest scoring MAG as the potential host [109]. Third, WIsH was used to assess likelihood of viral-host interactions between viral contigs and MAGs in this sample [110]. A *p*-value < 0.01 was considered significant, and null parameters were generated by creating a model from all RefSeq viruses with bacterial hosts, using the RefSeq database as of April 2020. As the above approaches rely on host genomes input by the user, a fourth method with the PHISdetector web server was used, which predicts likely hosts for phage contigs using a reference database [111]. The predicted host with the highest probability was taken, requiring at least two methods for prediction (e.g., from blast, WIsH, d2* matcher, or CRISPR). CoverM was used in ‘genome’ mode to estimate the coverage of both viral and bacterial genomes in the sample (RPKM) (https://github.com/wwood/CoverM [accessed on 5 November 2020]). Comparison of contigs from the three likely prophage (1461, 6630, 1064) associated with the *Planktothrix* microbial genome bin was accomplished through the web-based tool SimpleSynteny v. 1.4 [112]. The remaining bacteriophage contigs were blasted using KEGG GENES v. 94.1, a collection of gene databases that annotate based on NCBI RefSeq and GenBank resources [113]. Data, which includes the MAGs and the whole genome sequencing data, is available under BioProject ID PRJNA635673.

### 5.10. Statistical Analysis

Data was visualized and analyzed using R Studio for Mac OSX, v. 1.2.1335 (2015, RStudio, Boston, MA, USA). A repeated measures ANOVA with within-effect was conducted for fluorometry data. Raw data was depicted using line graphs over time. To make filament size comparisons, data was initially checked for normality using visual methods and employing a Shapiro–Wilk’s normality test. Statistical comparisons of the filament sizes between negative control and phage-inoculated samples were conducted using both an independent Welch two-sample *t*-test and a Wilcoxon rank sum test with continuity correction, based on the previous normality checks. A box plot was graphed, comparing the negative control and the phage-infected cultures. A *p*-value ≤ 0.05 was considered significant. Packages used include “tidyverse”, “rstatix”, “ggplot2”, and “ggpubr” [114,115,116,117].

## Figures and Tables

**Figure A1:**
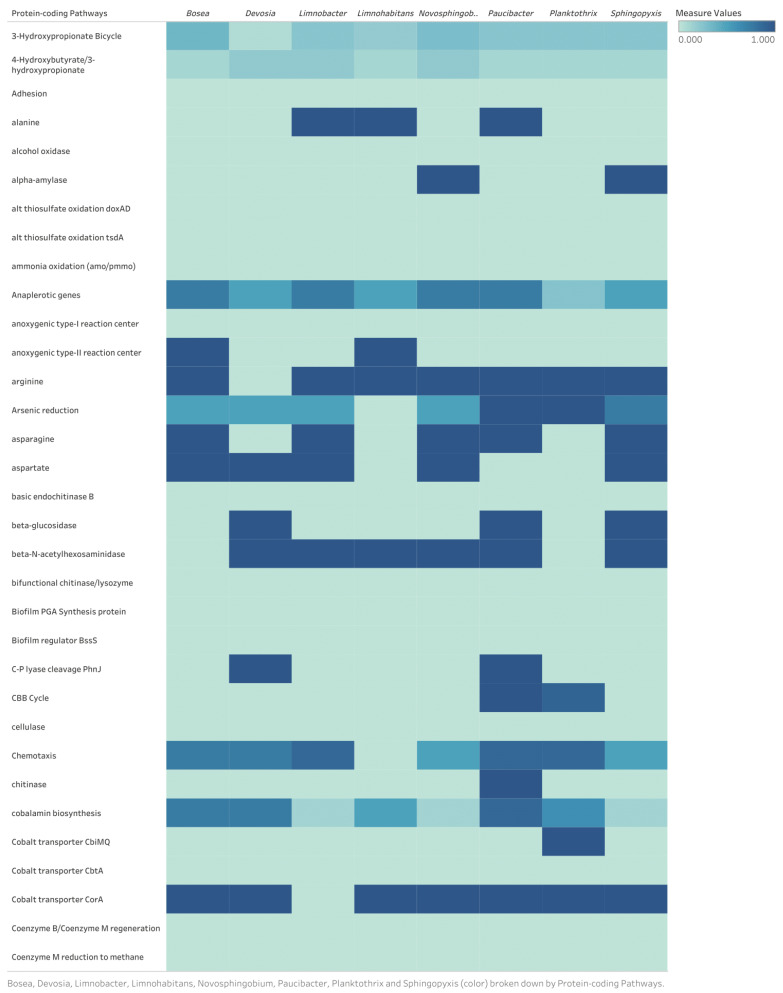

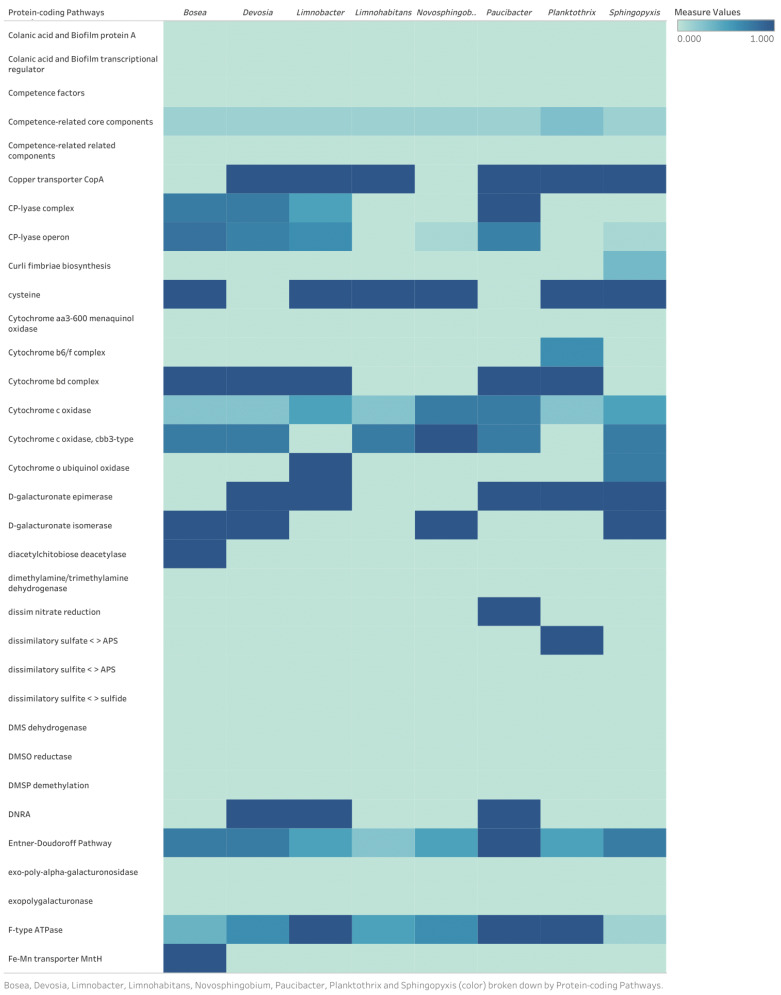

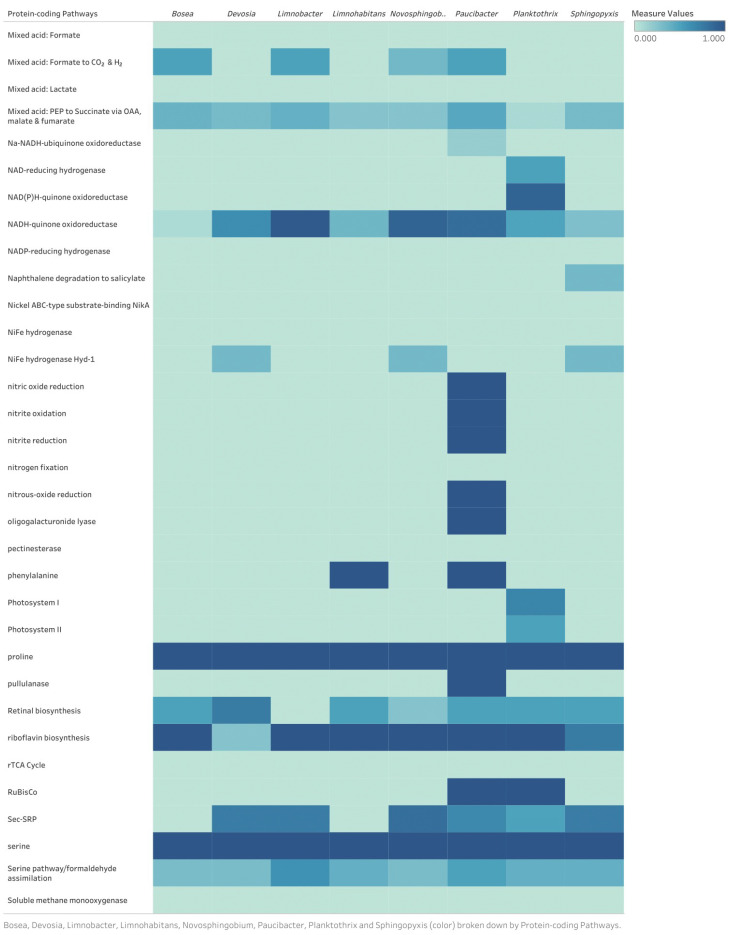

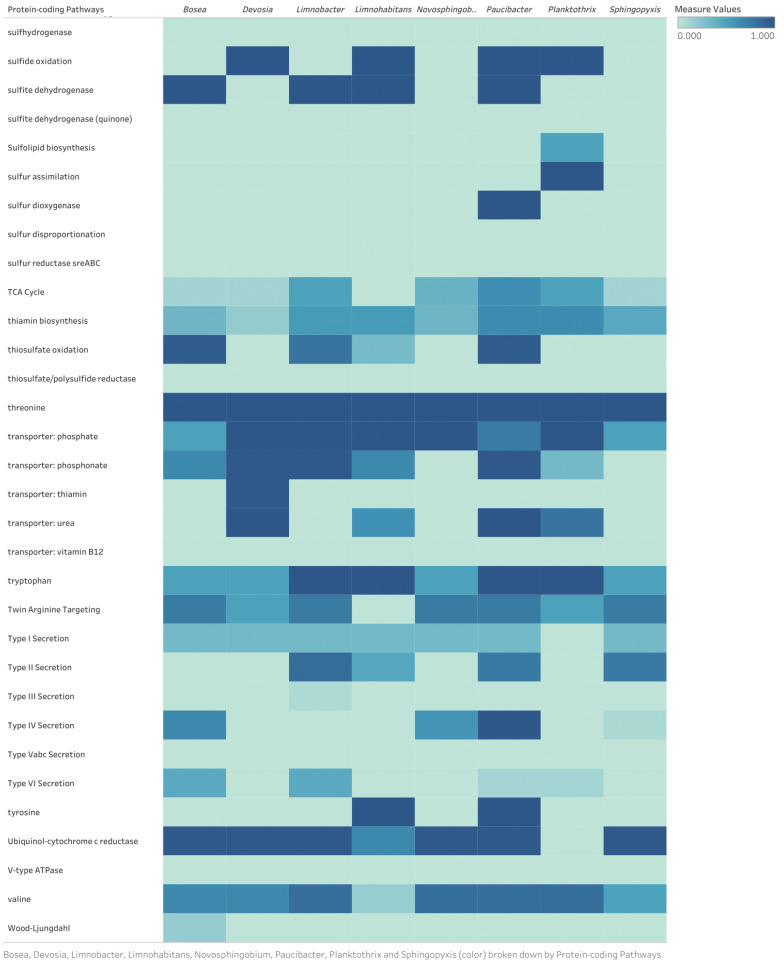

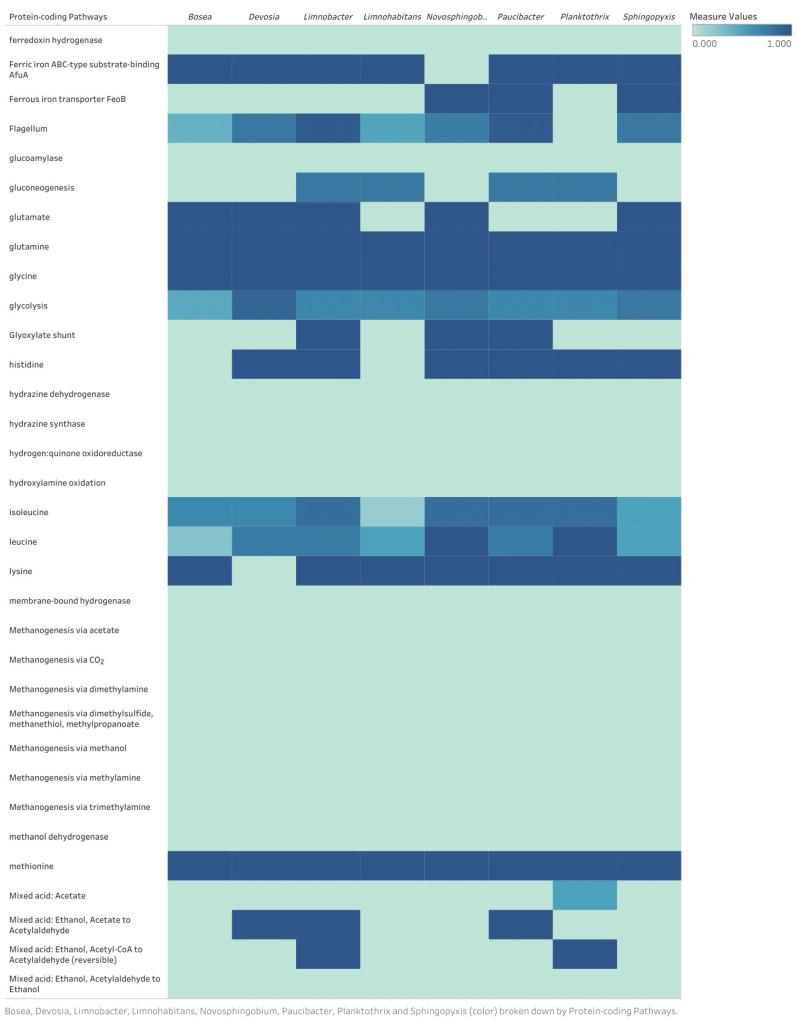
The full predicted protein-coding pathways heat map for the eight bacterial genomes.

**Figure A2:**
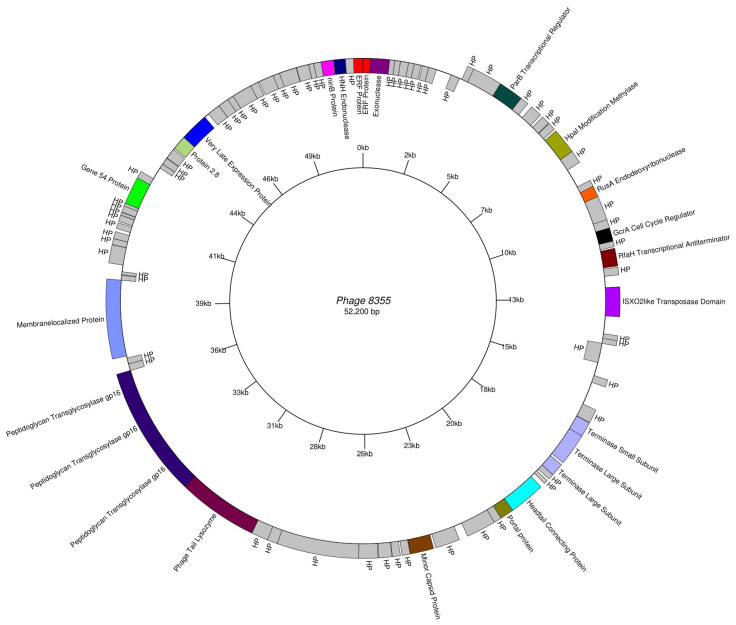
A high-quality, complete circular genome that was identified as one of the nineteen phages in the cyanobacteria culture. The genome contains 90 genes, 28% which are protein-coding.

## Data Availability

Data, which includes the MAGs and the whole genome sequencing data, is available under BioProject ID PRJNA635673.

## Supplementary Materials

The following supporting information can be downloaded at: https://www.mdpi.com/article/10.3390/toxins17090450/s1.

## Abbreviations

The following abbreviations are used in this manuscript:

WTRs	Water treatment residuals
DWTP	Drinking water treatment plant
HAB	Harmful algal bloom
MCs	Microcystins
SEM	Scanning electron microscopy
MAGs	Metagenome-assembled-genomes
VGFs	Viral genomic fragments
PCR	Polymerase chain reaction

## Appendix B

### Discussion of Identified Metabolic Pathways for Eight Bacterial Genomes

All of the bacteria contained important metabolic pathways related to amino acid and nucleotide metabolism, cell cycles and cell division, energy production, carbon fixation, transcription, and translation pathways. The variation in pathway completion likely relates to natural evolutionary divergences, as well as environmental pressures for different bacterial genera [118]. Most of the genomes contained an assortment of proteins essential for stress regulation, such as cellular oxidative stress reduction via cytochrome complexes, chemotaxis recognition for environmental stressors, electron-reduction enzymes that protect from overall toxicity, or trace element transporters for both growth and preventing intracellular toxicity. These pathways can be an important regulatory process in extreme environments [119]. For bacteria living in western Lake Erie near numerous industrial and commercial water outlets, as well as the bacteria concentrated into lagoons from a DWTP, the likelihood of exposure to stressors is high [120,121]. These environments contain numerous chemical or heavy metal stressors, requiring both adaptation and stress regulation pathways [122]. Moreover, the additional stress caused by phage infection likely activates many of these mechanisms, so having many different, unique pathways within the genome may support bacterial survival.

For the cyanobacteria *Planktothrix*, the target cyanobacteria for the phage infections, there were numerous identified metabolic pathways of interest. *P. agardhii* carried genes important for photosynthesis and energy conversion, such as Photosystem I and II, Cytochrome b6 complex, as well as sulfur uptake for cell stabilization during photosynthesis. These were expected, as these systems serve as their primary energy complexes [123,124]. Enzymes associated with major carbon fixation cycles (such as the Calvin cycle) are present in both the cyanobacteria genome as well as in one other bacterium, *Paucibacter*. In contrast, previous research on *Paucibacter* has described its minimal utilization of carbon sources for energy [68]. Further sequencing and testing of this strain would be necessary to better understand the primary processes utilized by this genus.

Most bacteria utilize the Type I, II, III, IV, V, and VI secretion pathways along with the Sec and TAT pathways for the movement of proteins, nucleic acids, and other molecules across membranes [125]. For all except *P. agardhii*, at least one of the Type I-VI secretion pathways was present. However, two other transport pathways, the traditional secretion pathway (Sec) and twin arginine targeting (TAT) pathways, were present in many bacteria, including *Planktothrix*. Pathways associated with the carbon-phosphate lyase operon were present in many of the bacteria, but again excluded *Planktothrix*. Numerous transmembrane protein systems were present in all of the identified bacteria. Cyanobacteria only utilize the latter two pathways, as they do not solely depend on the uptake of organic nutrients that are typically directed by Type I-VI secretion pathways [126]. The ability of bacteria like *Paucibacter* and *Sphingopyxis* to take up and metabolize secreted cyanobacterial toxins (in this case, the hepatotoxin MC that can be produced and released by *P. agardhii*) is an important metabolism in freshwater systems impacted by toxins. The likely competition within this environment requires such adaptations for survival through the utilization of molecules and nutrients from neighboring microorganisms.

All bacteria, excluding *Planktothrix* and *Limnohabitans*, contained C-P lyase operon-related genes. These genes allow for the cleavage of strong carbon-phosphate bonds of phosphonates to release methane, and are usually activated in phosphorus-starved bacteria [127]. Interestingly, most lakes are phosphorus-limited, but Lake Erie is known for high phosphorus levels due to agricultural and anthropogenic runoff [6,128]. Cyanobacteria, one of the dominant bacterial species during a bloom in a natural Lake Erie ecosystem, may be the primary microorganism for phosphorus metabolism and, therefore, cause high competition for this resource. Runoff events are becoming more intense and increasing in frequency, and with intermittent periods of drought, periods of limited phosphorus may cause this activation of C-P lyase pathways for other bacteria [129].

The enzyme beta-N-acetylhexosaminidase was present in almost all bacteria except *Bosea* and *Planktothrix*. This enzyme is especially important for the degradation, recycling, and reuse of membranes and cell-wall peptidoglycan [130]. As shown in the SEM images (Figure 4), the filaments of the cyanobacteria are broken apart, and extracellular matter is seen excreting from the cells. The other bacteria in the surrounding frame seem more abundant in the phage-inoculated sample compared to the control. It is possible that the increased growth of these bacteria may be due to the uptake and metabolism of the excreted nutrients from dying cyanobacteria filaments. Successful breakdown with enzymes like this one may allow for increased bacterial proliferation of other community members, as bloom collapse causes microbial community shifts and opportunistic species proliferate [131]. Together with the numerous transporters and active breakdown enzymes, the bacteria can utilize nutrients and continue to persist. This recycling of nutrients may be an important part of toxin degradation in these environments, but isolated experiments would be necessary to better understand this process.

Some general pathways excluded from nearly all of the genomes include nitrogen metabolism and fixation pathways, such as nitrogenase structural genes, ammonia oxidation, or nitrification pathways. Although it is well known that cyanobacteria and *P. agardhii* metabolize nitrogen as an energy source, no expected genes were identified in the *P. agardhii* genome [6,72]. Nitrogen-related pathways found particularly in soils and freshwater sediments were fully identified only in *Paucibacter*, *Limnobacter*, and *Devosia* [132,133]. Additionally, all genomes excluded any proteins associated with biofilm formation. This may be the result of under-characterized enzymes when compared to KEGG orthologs, or the incomplete reconstruction of some of the bacterial MAGs. Biofilms are more understudied and are quite complex, especially when looking for clear pathways with biofilm-related genes [55]. Characterization of genes responsible for biofilm formation in situ is likely needed to understand some of the phenotypes previously described in the phage-infection cultures.

Many pathways and functions in many of the identified bacteria were excluded from the *P. agardhii* genome. Proteins associated with flagella were found in all but the *Planktothrix* MAG. This is expected, as many bacteria use flagella for motility. This motility is important for numerous reasons, including stress response initiation and movement towards nutrients. *P. agardhii* is a naturally filamentous cyanobacterium that moves without the need for flagella.

## Appendix D

### Discussion of Identified Proteins in Three Phage Contigs

Six distinct proteins were identified in the three cyanophage contigs from the *Planktothrix* host genome, specifically those that are either genomic, structural, or phage-specific proteins. Structural proteins are related to phage assembly and are another important piece of viral infection. These proteins make up any of the three major structural units of a viral particle, such as the capsid head that protects the genetic material, the tail that allows for host attachment, and the head-tail connector that ensures safe travel between the two. Correct assembly ensures a successful infection of a host cell that can then go on to replicate and infect more host cells. A baseplate protein and a secretion system-like protein were two of the identified structural proteins. Baseplate proteins, located at the end of the tail sheath, are activated towards the later stages of viral replication and are important for host identification and specificity, as well as attachment and initiation of infection [134]. Type VI secretion system-like proteins are important for host bacteria as a protective mechanism against pathogens, and are just one of the many transport proteins mentioned previously. One study investigated phage genomes containing similar proteins and identified the similarities between the contractile tail mechanisms and the secretion system proteins [135]. The Type-VI secretion system creates a tube that attaches to a platform, similar to a baseplate protein, for molecule movement across the bacterial host membrane. Therefore, this may indicate that the identified protein is actually a tail-related protein associated with the contractile sheath, tail tube, or the baseplate.

Phage-specific proteins are proteins specifically found in phages and are directly related to infectivity. These proteins can be integrases that help viral DNA integration into the host genome, enzymes that repress or terminate host gene functions, chaperone portal proteins that aid in successful DNA passage through the tail sheath and successful viral DNA injection, and lysozymes that can degrade the host cell wall, as examples. Phage-specific infection proteins are some of the most important proteins that make a cyanophage lytic, allowing for continued viral replication and infection. DNA integrases, found in these genomes, initiate viral DNA integration into host DNA to then be replicated and activated for a successful infection [136].
